# Efficient and scalable patients clustering based on medical big data in cloud platform

**DOI:** 10.1186/s13677-022-00324-3

**Published:** 2022-09-24

**Authors:** Yongsheng Zhou, Majid Ghani Varzaneh

**Affiliations:** 1 Dongseo University Graduate School of Design, Busan, South Korea; 2 Shandong Provincial University Laboratory for Protected Horticulture, School of Art and Design, Weifang University of Science and Technology, Weifang, China; 3 Department of Electrical and Electronics Engineering, Shiraz University of Technology, Shiraz, Iran

**Keywords:** Cloud computing, Medical big data, Patients clustering, Data integration, Privacy

## Abstract

With the outbreak and popularity of COVID-19 pandemic worldwide, the volume of patients is increasing rapidly all over the world, which brings a big risk and challenge for the maintenance of public healthcare. In this situation, quick integration and analysis of the medical records of patients in a cloud platform are of positive and valuable significance for accurate recognition and scientific diagnosis of the healthy conditions of potential patients. However, due to the big volume of medical data of patients distributed in different platforms (e.g., multiple hospitals), how to integrate these data for patient clustering and analysis in a time-efficient and scalable manner in cloud platform is still a challenging task, while guaranteeing the capability of privacy-preservation. Motivated by this fact, a time-efficient, scalable and privacy-guaranteed patient clustering method in cloud platform is proposed in this work. At last, we demonstrate the competitive advantages of our method via a set of simulated experiments. Experiment results with competitive methods in current research literatures have proved the feasibility of our proposal.

## Introduction

With the outbreak of COVID-19 pandemic worldwide, the volume of patients who are infected by COVID-19 virus and other disasters (e.g., Diabetes Mellitus) is increasing rapidly, which brings a big risk and challenge for the healthcare of public all over the world [1–4]. In this situation, real-time collection and integration of the medical records of COVID-19 patients through a centralized cloud platform are of positive and valuable significance for accurate recognition and scientific diagnosis of the healthy conditions of patients [5–8]. Furthermore, through mining and analyzing the medical records of COVID-19 patients, global healthcare enterprises or organizations (e.g., World Health Organization) can quickly capture the variation tendency or mutation directions of the COVID-19 virus, so as to enact appropriate and fast treatment plans, alleviate patients’ pains and keep people much healthier [9–11].

However, the integration of the medical records of COVID-19 patients by a cloud platform is usually a challenging task due to the following reasons. (1) The COVID-19 patients are distributed all over the world; it is said there are COVID-19 patients in nearly 200 countries and regions. Therefore, the medical records of these distributed COVID-19 patients all over the world are hard to be integrated into a cloud platform because of the laws and regulations of different countries and regions [12, 13]. (2) The medical data of COVID-19 patients often contain partial privacy of patients, which impedes the effective and reliable data sharing between different medical platforms because certain patients are rather sensitive to personal privacy and often dare not release the private information to others [14–16]. (3) The COVID-19 patients are increasing rapidly in size; as a result, the corresponding medical data of patients are also growing very quickly, which places a heavy burden on the quick processing, mining and analyzing the big volume of medical data hosted in a cloud platform [17–19]. Moreover, the medical data are accumulated continuously with time elapsing, which raises a big challenge for the traditional medical data mining methods in cloud especially in terms of algorithm scalability.

Therefore, from the perspective of a cloud platform, how to integrate the massive and fast-growing medical data of COVID-19 patients in a time-efficient, scalable and privacy-preserving way is still a big challenge for integrating, processing and mining the medical data for providing better healthcare services to the public all over the world. Moreover, accurate clustering and division of the COVID-19 patients based on their medical data are of special significance and value to the accurate recognition and treatment of patients. For example, an anomaly or exception after patient clustering or division often indicates a valuable scientific finding in terms of healthy treatment. Therefore, it is of practical value or significance for global healthcare enterprises to investigate more effective and efficient patients clustering methods based on the medical data of COVID-19 patients registered in the cloud platform. However, the big volume and quick growth speed of medical data and the privacy disclosure concerns impede the realization of the above patient clustering goal. In view of the above challenges, this paper presents a novel efficient and scalable patient clustering method based on the historical medical data of patients in cloud environment, in a privacy-preserving way.

In summary, the major contributions of this paper are three-fold.

1. We recognize the importance and research significance of centralized patient clustering in cloud for global healthcare enterprises, especially in the big data context where a time-efficient, volume-scalable and privacy-preserving data integration strategy is required.
2. We introduce an effective ANN (Approximate Nearest Neighbor) discovery technique, i.e., Locality-Sensitive Hashing [20, 21] to perform time-efficient and scalable medical data integration and patient clustering in cloud environment in a privacy-preserving way.
3. A group of simulated experiments are enacted and deployed based on a popular dataset, which shows the feasibility and advantageous aspects of the proposed patient clustering method compared with other existing methods published in recent years.

We argue that our proposal can guarantee effective and efficient medical data integration especially in the big data context, due to the following two key points: (1) hash index is used in our proposed algorithm to secure the sensitive user information contained in medical data, which can reduce users’ privacy leakage concerns significantly; (2) hash index technique has been proven very time-efficient since its time complexity is approximately O(1); therefore, it is especially suitable for medical big data integration.

The reminder of this article is abbreviated as follows. In RELATED WORK section, we summarize the current research outcomes of the field. In MOTIVATION section, a concrete example is introduced to emphasize the research significance of this research work. In METHOD section, a novel time-efficient and scalable patient clustering method with privacy-preservation is put forward in cloud environment. In 5 section, experiment comparisons with related literatures are presented to prove the advantages of our proposal. In 6 section, we summarize the whole paper and discuss the possible research improvement directions in upcoming study.

## Related work

Medical data integration as well as patient clustering has been investigated for long decades. Next, we summarize the state-of-the-art research outcomes through the following two perspectives.

### Medical Data Protection

In [22], the authors propose an intelligent medical system in cloud based on blockchain technology, i.e., GuardHealth, which is mainly used for privacy protection and sharing of users’ medical data. Through effective and efficient privacy protection, GuardHealth improves the reliability of shared medical data significantly. Meanwhile, GuardHealth can also be used to realize the identification of malicious nodes and abnormal point detection to prevent unauthorized data sharing. Aiming at the privacy protection of medical data, the authors in [23] propose a promising privacy protection framework based on blockchain technology, i.e., MPBC. This framework has provisioned a new data storage method to alleviate the heavy burden brought by massive medical data that need to be shared and integrated for further data mining and analysis in cloud. Concretely, in the proposed MPBC framework, the authors introduce a federated learning mechanism to protect medical data by adding differential privacy noise into federated learning models. This way, sensitive user information in medical data is secured.

In [24], a deep learning-based intrusion detection method named deep belief network (DBN) model, is proposed, which is mainly used to detect and identify attacks and anomalies in the Internet of Things. By using the DBN model, security and privacy issues in the Internet of Things are well protected, and the method has achieved good results in terms of accuracy, recall, precision and other metrics. In [25], the authors recognize the challenge brought by privacy disclosure of medical data, because patients’ medical data are easy to leak and could damage patients’ privacy and hinder the development of medical undertakings in cloud. In this paper, the authors introduce a core technology in the Internet of Things - RFID. Through the security analysis and evaluation of the new scheme introduced in this paper, we can conclude that RFID can effectively solve the problem of privacy protection of medical data and prevent possible data leakage. In [26], the authors propose a new framework to realize data sharing and a new data access mechanism for safe storage and transmission of medical data. The method can accurately obtain patient information while ensuring patient data privacy.

### Patient Clustering & Anomaly Detection

In [27], the authors discuss the patient clustering and anomaly detection issues based on medical image data analysis and processing in cloud. In concrete, this paper presents the classification of the literatures about related applications, sums up the advantages and disadvantages of different medical data processing schemes, highlights their important results, and summarizes experience and lessons. Meanwhile, several suggestions on how to analyze the medical image data for anomaly detection are also given. In [28], the authors use medical data to make disease classification. Besides, patient clustering and data analysis with anomaly detection algorithms are presented, and the development of existing methods of disease prevention and application situation are also discussed.

In [29], the authors propose an anomaly detection framework based on generative adversarial network, to solve various medical problems by mining the annotated large-scale medical data in cloud. The framework is developed to address the limitations of a diagnosis process that is difficult for diseases with relatively low incidence. Through a sparse and constrained generative adversarial network proposed in this paper, we can make accurate disease screening. At the same time, authors also propose to use an abnormal activation map to display the heat map of lesions. In [30], the authors propose a generative admission network for unsupervised medical anomaly detection. Because unsupervised learning can find some invisible anomalies, 2D or 3D medical images can be constructed through unsupervised learning to detect outliers. In [31], through unsupervised learning that can be used when it is not necessary to mark large-scale medical data as well as their characteristics, a variational context of coding automatic encoder (ceVAE) framework based on unsupervised learning is introduced. Typically, the framework implements the reconstruction by integrating the density anomaly scores, through which we can achieve satisfactory results in recognizing possible anomalies or exceptions.

With the above analysis, we can find that existing literatures still fall short in achieving time-efficient and scalable patient clustering while guaranteeing sensitive user privacy, while computational cost and user privacy are both important in big data applications [32–40]. Inspired by this shortcoming, we propose a new solution in the following sections.

## Motivation

**Fig. 1:**
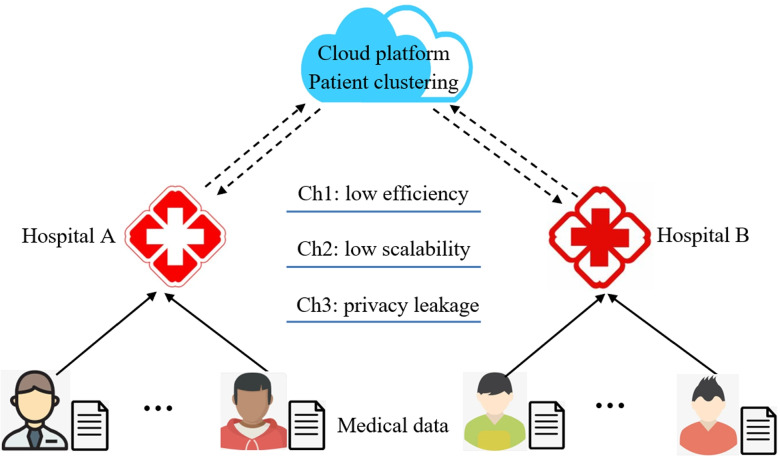
An example of patient clustering based on distributed medical data in cloud platform

We present a concrete example in Fig. 1 to better clarify the motivation of this paper. In Fig. 1, some patients as well as their medical data are hosted in Hospital A, while other patients as well as their medical data are hosted in Hospital B. For comprehensive medical data analysis of global healthcare enterprises, we need to integrate the medical data of patients distributed in Hospitals A and B into a central cloud platform to make accurate patient clustering and division. However, the above integration and clustering process often confronts with three challenges: Ch1: due to the big volume of medical data of patients in different hospitals, much time is often required to pre-process, integrate and cluster the integrated medical data, which probably leads to low time efficiency; Ch2: the medical data of patients in different hospitals are accumulated with time, which calls for additional computational time to update the patient clustering model, i.e., the scalability is often low; Ch3: since the medical data of patients in different hospitals need to be transmitted to the central cloud platform, patients’ sensitive data contained in medical data are probably disclosed to the third party, which further impedes the medical data sharing willingness of patients and hospitals.

In view of these challenges, we bring forth a novel patient clustering method based on medical data of patients in different hospitals, in a time-efficient, volume-scalable and privacy-preserving manner through a cloud platform. The detailed procedure of our proposed method is described in the following section.

## Method

Next, we introduce Locality-Sensitive Hashing into time-efficient, scalable and privacy-preserving patient clustering process and bring forth a novel Patient Clustering Method in cloud based on medical data distributed in different platforms, named PCM. For better understanding and discussion in the subsequent paragraphs, we make the following formalization: assume there are m patients in P-Set = {$p_1$,...,$p_m$},n disasters in D-Set = {$d_1$,...,$d_n$}, matrix M in Eq. (1) depicts the patients’ health monitoring data on different disasters: $a_{i,j}$ denotes patient $p_i$’s health data on disaster $d_j$ $(1 \le i \le m, 1 \le j \le n)$. Here, M is an m*n matrix as shown in Eq. (1).

1 $$\begin{aligned} M = \begin{array}{ll}&\begin{array}{ccc} \quad \ \ \, d_1 &{} \cdots &{} d_n \end{array}\\ \begin{array}{l} p_1 \\ \vdots \\ p_m \end{array} &{} \left[ \begin{array}{lll} a_{1, 1}&{}\cdots &{}a_{1, n} \\ \vdots &{}\ddots &{}\vdots \\ a_{m, 1}&{}\cdots &{}a_{m, n}\end{array}\right] \end{array} \end{aligned}$$

Next, we need to cluster the m patients into multiple groups based on the medical data in matrix M, in a time-efficient, scalable and privacy-preserving way. In concrete, the major procedure of the PCM method is described in detail as follows.

**Step 1: Patient index generation based on medical data.**

Next, we generate the index of each patient based on the patient’s medical data in matrix M registered in a cloud platform. In concrete, as Eq. (1) shows, each patient $p_i$’s medical data are represented by a vector V($p_i$) = ($a_{i,1}$,...,$a_{i,n}$). Next, we generate another vector V = ($v_1$,...,$v_n$) according to Eq. (2). In other words, each entry of vector V is a random data between -1 and 1. Thus, with vector V($p_i$) and vector V, a new data w($p_i$) is obtained by Eq. (3) and (4).

2 $$\begin{aligned} v_i = Rand~(-1,1) \end{aligned}$$

3 $$\begin{aligned} if~ V(p_i)*V > 0, then~ w(p_i) = 1 \end{aligned}$$

4 $$\begin{aligned} if~ V(p_i)*V \le 0, then~ w(p_i) = 0 \end{aligned}$$

For each patient $$p_i$$, we execute Eq. (2)-(4) c times (c = 2, 3...) and then we get c 0/1 values corresponding to patient $$p_i$$ : $$w_1$$($$p_i$$),...,$$w_c$$($$p_i$$). Next, we merge the c 0/1values into a new Boolean value W($$p_i$$) = ($$w_1$$($$p_i$$)$$w_2$$($$p_i$$)...$$w_c$$($$p_i$$)$$)_B$$. For example, if c = 3 and $$w_1$$($$p_i$$) = 1, $$w_2$$($$p_i$$) = 1, $$w_3$$($$p_i$$) = 0, then W($$p_i$$) = (110$$)_B$$. For simplicity of subsequent specifications, we transform W($$p_i$$) from a Boolean value to a Decimal value. For example, W($$p_i$$) = (110$$)_B$$ = 6. Thus, through the above calculation, matrix M in Eq. (1) is converted into the following matrix M* in Eq. (5).

5 $$\begin{aligned} M^* = \begin{array}{ll} \begin{array}{c} p_1 \\ \vdots \\ p_m \end{array} &{} \left[ \begin{array}{c} \ W(p_1) \\ \vdots \\ \ W(p_m) \end{array}\right] \end{array} \end{aligned}$$

Next, we execute the operations in Eq. (5) r times (r = 2, 3...) and then for each patient $p_i$, we get r Decimal values: $W_1$($p_i$),...,$W_r$($p_i$). Then the matrix $M^*$ in Eq. (5) is converted into the following matrix $M^\#$ in Eq. (6). Then according to the hash projection rule of Locality-Sensitive Hashing, each row vector in matrix $M^\#$ is the index for the patient corresponding to the row. For example, we consider the first row of matrix $M^\#$: h($p_1$) = ($W_1$($p_1$),...,$W_r$($p_1$)) is the index of patient $p_1$, and so on. This way, we can convert the medical data of each patient $p_i$ into a corresponding index h($p_i$),i.e., Eq. (7) holds.

6 $$\begin{aligned} M^\# = \begin{array}{ll} \begin{array}{c} p_1 \\ \vdots \\ p_m \end{array} &{} \left[ \begin{array}{c} \left( W_1(p_1) \cdots W_r(p_1) \right) \\ \vdots \\ \left( W_1(p_m) \cdots W_r(p_m) \right) \end{array}\right] \end{array} \end{aligned}$$

7 $$\begin{aligned} M^\# = \begin{array}{ll} \begin{array}{c} p_1 \\ \vdots \\ p_m \end{array} &{} \left[ \begin{array}{c} \ h(p_1) \\ \vdots \\ \ h(p_m) \end{array}\right] \end{array} \end{aligned}$$

Then we put the projections from patients $p_i$ to their corresponding indexes h($p_i$) into a table (i.e., index table In-T), as illustrated in Table 1. According to the basic rule of Locality-Sensitive Hashing, if h($p_i$) = h($p_j$) holds, then the two patients $p_i$ and $p_j$ are close with high probability. However, the above similar patient discovery way is a bit simple and straightforward and sometimes not correct because of the probability-based nature of Locality-Sensitive Hashing. In other words, False-negative or False-positive cases are inevitable. To tackle this issue, one index table is often not enough and therefore, multiple index tables are necessary to be created. Next, we repeat the index table generation process in Table 1 k times (k = 2, 3, ...) and obtain k index tables (In-$T_1$,...,In-$T_k$) as illustrated in Table 2.

**Table 1:** An index table of patients

Patient	Index
$p_1$	$h(p_1)$
$p_2$	$h(p_2)$
...	...
$p_m$	$h(p_m)$

**Table 2:** k index tables of patients

Patient	$In-T_1$	...	$In-T_k$
	Index	Index	Index
$p_1$	$h_1(p_1)$	...	$h_k(p_1)$
$p_2$	$h_1(p_2)$	...	$h_k(p_2)$
...	...	...	...
$p_m$	$h_1(p_m)$	...	$h_k(p_m)$

**Step 2: Patient clustering based on patient index.**

In Step1, we have got k index tables for m patients: In-$T_1$,...,In-$T_k$.Next, we cluster the m patients into different groups according to In-$T_1$,...,In-$T_k$.In concrete, the clustering process is formalized as in Eq. (8). More intuitively, for two patients $p_i$ and $p_j$,their similarity degree (denoted by S($p_i$,$p_j$)) is equal to 1 if their indexes are equal in any of the k index tables: In-$T_1$,...,In-$T_k$.Next, the patients whose similarity degree is equal to 1 are put into the same group or cluster. This way, the m patients are successfully clustered into different groups. Since the index tables of patients are produced offline and the time complexity is close to 0, the above patient clustering process if often time-efficient and scalable (time complexity is O(1)). Moreover, we only use patient indexes in Table 2 to achieve the clustering goal without revealing the real medical data in matrix M in Eq. (1); therefore, little privacy is disclosed in the clustering process. In other words, we can cluster the patients successfully in a privacy-preserving way.

8 $$\begin{aligned} S(p_i,p_j) = 1 ~ iff~ h_x(p_i) = h_x(p_j)~holds~in~any~In-T_x(x = 1,2,...,k) \end{aligned}$$

In summary, our proposal can guarantee effective and efficient medical data integration especially in the big data context because of the following two reasons: (1) hash index is used in our proposed algorithm to secure the sensitive user information contained in medical data, which can reduce users’ privacy leakage concerns significantly; (2) hash index technique has been proven very time-efficient since its time complexity is approximately O(1); therefore, it is especially suitable for medical big data integration.

We use the following pseudocode to formulate the PCM algorithm.

**Figure:**
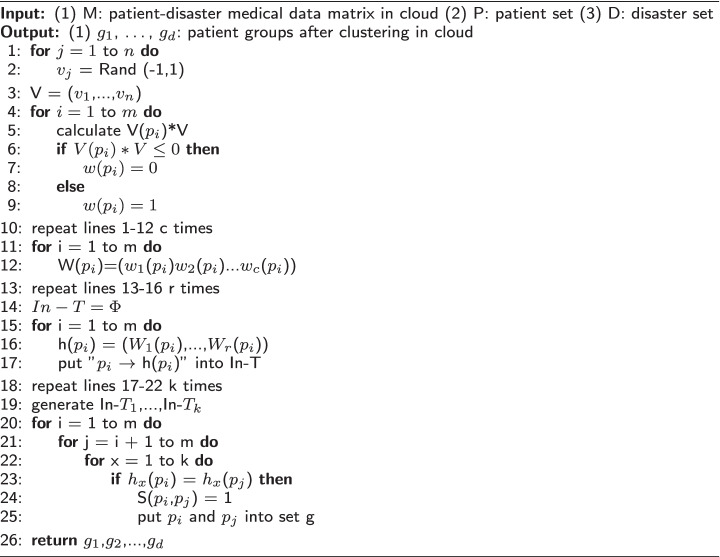
**Algorithm 1** PCM(M,P,D)

## Evaluation

**Setting**

1. Hardware: Intel(R) Core(TM) i7-6500U CPU @ 2.50 GHz, 16.0 GB RAM.
2. Software: Win 10, Python 3.0.
3. Dataset: we use WS-DREAM dataset for simulated experiments and each experiment is run 50 times and we register their average performances for final display.
4. Compared methods: SerRe$c_{distri-LSH}$ [41] and UPCC [17].
5. Metrics: MAE, RMSE, Time cost.

**Results**

(1) MAE comparison.

**Fig. 2:**
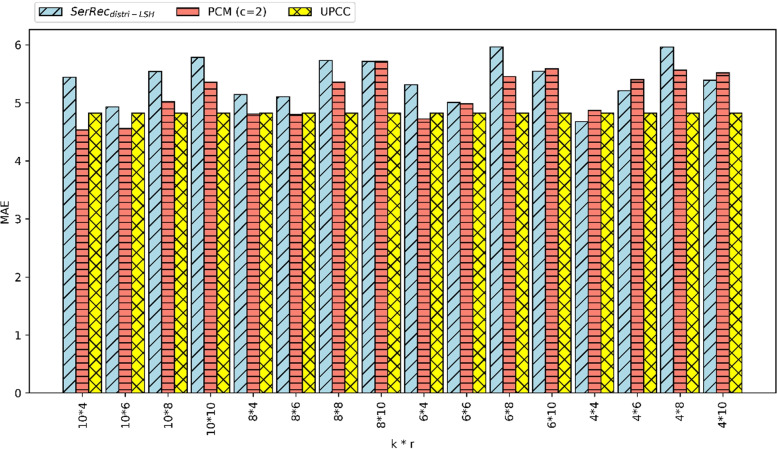
MAE of three methods

MAE is a common and popular criterion to measure the prediction accuracy is big data applications [42–49]. Here, the MAE performances of three methods are measured and compared. Parameter settings are as follows: m = 300, n = 5000, c = 2, k = 4, 6, 8, 10, r = 4, 6, 8, 10. The parameter settings of m, n, k and r in the experiment evaluation section are based on experience. In concrete, we have tested several sets of parameters k and r in the experiments and found that m = 300, n = 5000, k = 4, 6, 8, 10 and r = 4, 6, 8, 10 are appropriate settings to observe the performances of our proposal. Comparison reports are presented in Fig. 2 where UPCC is the baseline method whose MAE is the smallest (smaller MAE indicates higher clustering accuracy). Regarding SerRe$c_{distri-LSH}$ and our PCM (c = 2), PCM’s MAE is often smaller than that of SerRe$c_{distri-LSH}$, which indicates that in most cases PCM’s clustering accuracy is higher than SerRe$c_{distri-LSH}$. The reason is that we introduce a new factor of parameter c, which strengthens the clustering performances more or less. Therefore, PCM can achieve better clustering performances (especially the clustering accuracy) than SerRe$c_{distri-LSH}$. Although UPCC performs better than PCM in certain situations, UPCC cannot protect user privacy well while our PCM method can.

(2) RMSE comparison.

**Fig. 3:**
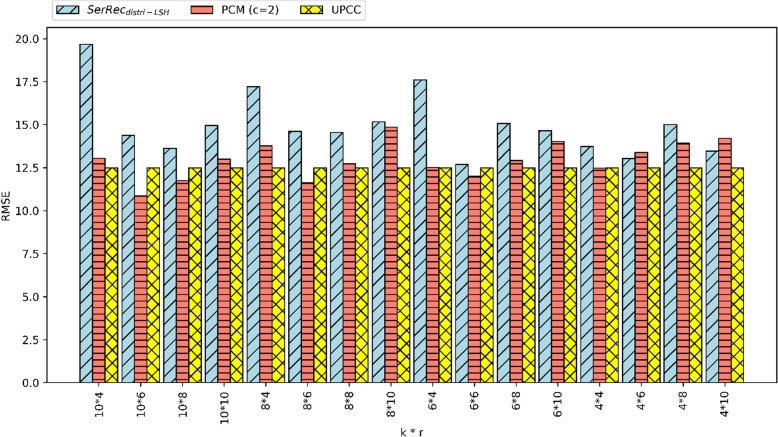
RMSE of three methods

Here, we use another metric RMSE to evaluate the clustering accuracy of different methods and parameters are set as follows: m = 300, n = 5000, c = 2, k = 4, 6, 8, 10, r = 4, 6, 8, 10. Concrete experiment results are reported in Fig. 3 where UPCC is the baseline method whose RMSE is the smallest (smaller RMSE often means higher cluster accuracy). Regarding SerRe$c_{distri-LSH}$ and our PCM (c = 2), PCM’s RMSE is often smaller than that of SerRe$c_{distri-LSH}$, which means that in most cases PCM’s clustering accuracy is better than SerRe$c_{distri-LSH}$. The reason is the same as that we analyzed in Fig. 2, i.e., we introduce a new factor of parameter c, which improves the clustering performance considerably. Therefore, PCM can achieve better clustering effect than SerRe$c_{distri-LSH}$. Although UPCC performs better than PCM in partial cases, UPCC cannot protect user privacy well while PCM can.

(3) Time cost comparison.

**Fig. 4:**
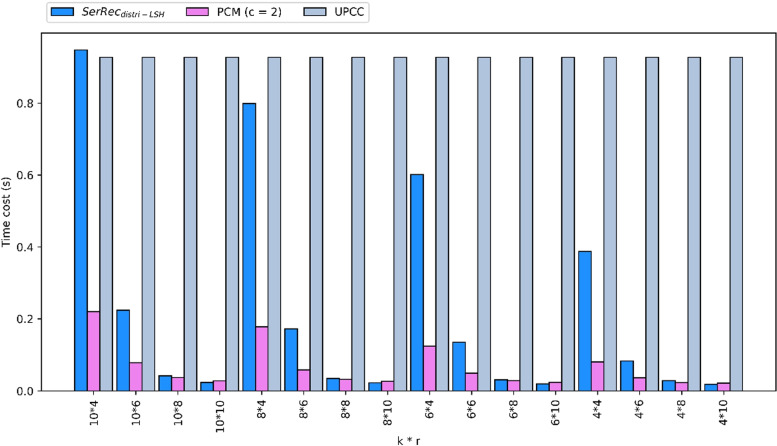
Time cost of three methods

Computational cost is an important metric that plays a key role in big data systems [50–53]. Motivated by this fact, we measure the consumed time cost of three methods. Concrete parameters are set as follows: m = 300, n = 5000, c = 2, k = 4, 6, 8, 10, r = 4, 6, 8, 10. Concrete experiment results are shown in Fig. 4. As can be seen from the results, UPCC consumed the most time since it involves massive calculation operations of user similarity based on Pearson Correlation Coefficients. For both SerRe$c_{distri-LSH}$ and our PCM, their time costs are both small because they both use offline indexes to make similar user clustering. Moreover, the time complexity of our PCM is better than that of SerRe$c_{distri-LSH}$ because in PCM, we use improved Locality-Sensitive Hashing to create indexes and as a result, only fewer similar users are finally returned for subsequent clustering. Correspondingly, time cost is reduced considerably.

## Conclusions

With the outbreak of COVID-19 pandemic worldwide, the volume of patients is increasing rapidly, which brings a big risk and challenge for the healthcare of public all over the world. In this situation, quick integration and analysis of the medical records of patients are of positive and valuable significance for accurate recognition and scientific diagnosis of the healthy conditions of patients. However, due to the big volume of medical data of patients distributed in different platforms (e.g., hospitals), how to integrate these data efficiently for patient clustering and analysis is still a challenging task, while guaranteeing privacy-preservation and high scalability. Motivated by this fact, a time-efficient, scalable and privacy-guaranteed patient clustering method is proposed in this work. Our proposal can guarantee effective and efficient medical data integration especially in the big data context, due to the following two key points: (1) hash index is used in our proposed algorithm to secure the sensitive user information contained in medical data, which can reduce users’ privacy leakage concerns significantly; (2) hash index technique has been proven very time-efficient since its time complexity is approximately O(1); therefore, it is especially suitable for medical big data integration. At last, we demonstrate the competitive advantages of our method via a set of simulated experiments.

However, there are several limitations in this work. First, we assume that the medical data are of a fixed type, while data types are of multiple and varied in the big data context [54–56]. Therefore, it is necessary to improve the PCM method by incorporating different types of medical data. Moreover, medical data are often not static, but associated with many context factors such as time, location and so on [57–60]. Therefore, in the future study, we will continue to optimize our proposed PCM method by considering multiple context factors. Finally, the analysis and processing of big data often call for a considerable amount of computing resources as well as effective computing offloading strategies and energy-saving technologies [61–64]. In the upcoming research work, we will continue to study more economic and green resolutions.

## Data Availability

WS-DREAM: http://wsdream.github.io/.

## Abbreviations

ANN: Approximate Nearest Neighbor
MPBC: Medical data Protection based on BlockChain
PCM: Patient Clustering Method
UPCC: User-based Pearson Correlation Coefficient
DBN: Deep Belief Network

## References

[1] Kaixin Li, Jie Zhao, Jintao Hu, et al (2022) Dynamic Energy Efficient Task Offloading and Resource Allocation for NOMA-enabled IoT in Smart Buildings and Environment. Build Environ. 10.1016/j.buildenv.2022.109513

[2] Gupta VK, Gupta A, Kumar D, Sardana A (2021). Prediction of COVID-19 confirmed, death, and cured cases in India using random forest model. Big Data Min Anal.

[3] Yang Y (2015). Attribute-based data retrieval with semantic keyword search for e-health cloud. J Cloud Comput.

[4] Xu X, Tian H, Zhang X, Qi L, He Q, Dou W (2022). DisCOV: distributed COVID-19 detection on X-ray images with edge-cloud collaboration. IEEE Trans Serv Comput.

[5] Kumari R, Kumar S, Poonia RC, Singh V, Raja L, Bhatnagar V (2021). Analysis and predictions of spread, recovery, and death caused by COVID-19 in India. Big Data Min Anal.

[6] Uslu BÇ, Okay E, Dursun E (2020). Analysis of factors affecting IoT-based smart hospital design. J Cloud Comput.

[7] Pang J, Huang Y, Xie Z, Li J, Cai Z (2021). Collaborative city digital twin for the COVID-19 pandemic: A federated learning solution. Tsinghua Sci Technol.

[8] Singh KK, Singh A (2021). Diagnosis of COVID-19 from chest X-ray images using wavelets-based depthwise convolution network. Big Data Min Anal.

[9] Agarwal A, Sharma S, Kumar V, Kaur M (2021). Effect of E-learning on public health and environment during COVID-19 lockdown. Big Data Min Anal.

[10] Liu Y, Song Z, Xu X, Rafique W, Zhang X, Shen J (2022). Bidirectional GRU networks-based next POI category prediction for healthcare. Int J Intell Syst.

[11] Kong L, Wang L, Gong W, Yan C, Duan Y, Qi L (2021) LSH-aware multitype health data prediction with privacy preservation in edge environment. World Wide Web 1–16. 10.1007/s11280-021-00941-z

[12] Shao Q, Yu R, Zhao H, Liu C, Zhang M, Song H (2021). Toward intelligent financial advisors for identifying potential clients: a multitask perspective. Big Data Min Anal.

[13] Yuan Q, Wang D, Zhao Y, Sang Y, Wang F, Liu Y (2021). Privacy-aware examination results ranking for the balance between teachers and mothers. Tsinghua Sci Technol.

[14] Wang F, Li G, Wang Y, Rafique W, Khosravi MR, Liu G, et al (2022) Privacy-aware traffic flow prediction based on multi-party sensor data with zero trust in smart city. ACM Trans Internet Technol (TOIT). 10.1145/3511904

[15] Zhang K, Tian Z, Cai Z, Seo D (2021). Link-privacy preserving graph embedding data publication with adversarial learning. Tsinghua Sci Technol.

[16] Zheng X, Zhang L, Li K, Zeng X (2021). Efficient publication of distributed and overlapping graph data under differential privacy. Tsinghua Sci Technol.

[17] Bouras MA, Farha F, Ning H (2020). Convergence of computing, communication, and caching in Internet of Things. Intell Converged Netw.

[18] Zhou X, Li Y, Liang W (2020). CNN-RNN based intelligent recommendation for online medical pre-diagnosis support. IEEE/ACM Trans Comput Biol Bioinforma.

[19] Zhou X, Liang W, Kevin I, Wang K, Yang LT (2020). Deep correlation mining based on hierarchical hybrid networks for heterogeneous big data recommendations. IEEE Trans Soc Syst.

[20] Qi L, Hu C, Zhang X, Khosravi MR, Sharma S, Pang S (2021). Privacy-aware data fusion and prediction with spatial-temporal context for smart city industrial environment. IEEE Trans Ind Inform.

[21] Qi L, Yang Y, Zhou X, Rafique W, Ma J (2021) Fast Anomaly Identification Based on Multi-Aspect Data Streams for Intelligent Intrusion Detection Toward Secure Industry 4.0. IEEE Trans Ind Inform. 10.1109/TII.2021.3139363

[22] Wang Z, Luo N, Zhou P (2020). GuardHealth: Blockchain empowered secure data management and Graph Convolutional Network enabled anomaly detection in smart healthcare. J Parallel Distrib Comput.

[23] Zhang H, Li G, Zhang Y, Gai K, Qiu M (2021) Blockchain-based privacy-preserving medical data sharing scheme using federated learning. In: International Conference on Knowledge Science, Engineering and Management. Springer, p 634–646

[24] Manimurugan S, Al-Mutairi S, Aborokbah MM, Chilamkurti N, Ganesan S, Patan R (2020). Effective attack detection in internet of medical things smart environment using a deep belief neural network. IEEE Access.

[25] Fan K, Jiang W, Li H, Yang Y (2018). Lightweight RFID protocol for medical privacy protection in IoT. IEEE Trans Ind Inform.

[26] Fang L, Yin C, Zhu J, Ge C, Tanveer M, Jolfaei A (2020). Privacy protection for medical data sharing in smart healthcare. ACM Trans Multimed Comput Commun Appl (TOMM).

[27] Tschuchnig ME, Gadermayr M (2022) Anomaly Detection in Medical Imaging-A Mini Review. Data Sci Anal Appl 33–38

[28] Razzak MI, Imran M, Xu G (2020). Big data analytics for preventive medicine. Neural Comput & Applic.

[29] Zhou K, Gao S, Cheng J, Gu Z, Fu H, Tu Z, et al (2020) Sparse-gan: Sparsity-constrained generative adversarial network for anomaly detection in retinal oct image. In: 2020 IEEE 17th International Symposium on Biomedical Imaging (ISBI). IEEE, p 1227–1231

[30] Han C, Rundo L, Murao K, Noguchi T, Shimahara Y, Milacski ZÁ (2021). MADGAN: Unsupervised medical anomaly detection GAN using multiple adjacent brain MRI slice reconstruction. BMC Bioinformatics.

[31] Zimmerer D, Kohl SA, Petersen J, Isensee F, Maier-Hein KH (2018) Context-encoding variational autoencoder for unsupervised anomaly detection. arXiv preprint arXiv:1812.05941

[32] Zhou X, Liang W, Li W, Yan K, Shimizu S, Wang KIK (2022). Hierarchical Adversarial Attacks Against Graph-Neural-Network-Based IoT Network Intrusion Detection System. IEEE Internet Things J.

[33] Huang J, Tong Z, Feng Z (2022) Geographical POI recommendation for Internet of Things: A federated learning approach using matrix factorization. Int J Commun Syst. 10.1002/dac.5161

[34] Dai H, Xu Y, Chen G, Dou W, Tian C, Wu X (2022). ROSE: Robustly Safe Charging for Wireless Power Transfer. IEEE Trans Mob Comput.

[35] Chen Y, Zhao F, Lu Y, Chen X (2021) Dynamic task offloading for mobile edge computing with hybrid energy supply. Tsinghua Sci Technol. 10.26599/TST.2021.9010050

[36] Cai Z, He Z, Guan X, Li Y (2018). Collective Data-Sanitization for Preventing Sensitive Information Inference Attacks in Social Networks. IEEE Trans Dependable Secure Comput.

[37] Xu Y, Liu Z, Zhang C, Ren J, Zhang Y, Shen X (2022). Blockchain-Based Trustworthy Energy Dispatching Approach for High Renewable Energy Penetrated Power Systems. IEEE Internet Things J.

[38] Li T, Li C, Luo J, Song L (2020). Wireless recommendations for Internet of vehicles: Recent advances, challenges, and opportunities. Intell Converged Netw.

[39] Qi L, Lin W, Zhang X, Dou W, Xu X, Chen J (2022) A Correlation Graph based Approach for Personalized and Compatible Web APIs Recommendation in Mobile APP Development. IEEE Trans Knowl Data Eng 1. 10.1109/TKDE.2022.3168611

[40] Chen Y, Gu W, Li K (2022) Dynamic task offloading for Internet of Things in mobile edge computing via deep reinforcement learning. Int J Commun Syst. 10.1002/dac.5154

[41] Qi L, Zhang X, Dou W, Ni Q (2017). A distributed locality-sensitive hashing-based approach for cloud service recommendation from multi-source data. IEEE J Sel Areas Commun.

[42] Zhou X, Yang X, Ma J, Wang KIK (2022). Energy-Efficient Smart Routing Based on Link Correlation Mining for Wireless Edge Computing in IoT. IEEE Internet Things J.

[43] Gu R, Chen Y, Liu S, Dai H, Chen G, Zhang K (2022). Liquid: Intelligent Resource Estimation and Network-Efficient Scheduling for Deep Learning Jobs on Distributed GPU Clusters. IEEE Trans Parallel Distrib Syst.

[44] Cai Z, Zheng X (2020). A Private and Efficient Mechanism for Data Uploading in Smart Cyber-Physical Systems. IEEE Trans Netw Sci Eng.

[45] Xu Y, Zhang C, Wang G, Qin Z, Zeng Q (2021). A Blockchain-Enabled Deduplicatable Data Auditing Mechanism for Network Storage Services. IEEE Trans Emerg Top Comput.

[46] Zhou X, Xu X, Liang W, Zeng Z, Yan Z (2021). Deep-Learning-Enhanced Multitarget Detection for End-Edge-Cloud Surveillance in Smart IoT. IEEE Internet Things J.

[47] Dai H, Wu C, Wang X, Dou W, Liu Y (2020) Placing Wireless Chargers with Limited Mobility. In: IEEE INFOCOM 2020 - IEEE Conference on Computer Communications. pp 2056–2065. 10.1109/INFOCOM41043.2020.9155356

[48] Cai Z, He Z (2019) Trading Private Range Counting over Big IoT Data. In: 2019 IEEE 39th International Conference on Distributed Computing Systems (ICDCS). pp 144–153. 10.1109/ICDCS.2019.00023

[49] Zhang C, Xu Y, Hu Y, Wu J, Ren J, Zhang Y (2021) A Blockchain-Based Multi-Cloud Storage Data Auditing Scheme to Locate Faults. IEEE Trans Cloud Comput 1. 10.1109/TCC.2021.3057771

[50] Huang J, Lv B, Wu Y (2022). Dynamic Admission Control and Resource Allocation for Mobile Edge Computing Enabled Small Cell Network. IEEE Trans Veh Technol.

[51] Catlett C, Beckman P, Ferrier N, Nusbaum H, Papka ME, Berman MG (2020). Measuring cities with software-defined sensors. J Soc Comput.

[52] Chen Y, Liu Z, Zhang Y, el al, (2021) Deep reinforcement learning-based dynamic resource management for mobile edge computing in industrial internet of things. IEEE Trans Ind Inform 17(7):4925–4934

[53] Zhi P, Zhao R, Zhou H, Zhou Y, Ling N, Zhou Q (2021). Analysis on the Development Status of Intelligent and Connected Vehicle Test Site. Intell Converged Netw.

[54] Zhou J, Cao K, Zhou X, Chen M, Wei T, Hu S (2022). Throughput-Conscious Energy Allocation and Reliability-Aware Task Assignment for Renewable Powered In-Situ Server Systems. IEEE Trans Comput Aided Des Integr Circ Syst.

[55] Gu R, Zhang K, Xu Z, Che Y, Fan B, Hou H, et al (2022) Fluid: Dataset Abstraction and Elastic Acceleration for Cloud-native Deep Learning Training Jobs. In: 2022 IEEE 38th International Conference on Data Engineering (ICDE). pp 2182–2195. 10.1109/ICDE53745.2022.00209

[56] Wang Y, Gao Y, Li Y, Tong X (2020) A worker-selection incentive mechanism for optimizing platform-centric mobile crowdsourcing systems. Comput Netw 171:107144. 10.1016/j.comnet.2020.107144

[57] Xu Y, Ren J, Zhang Y, Zhang C, Shen B, Zhang Y (2020). Blockchain Empowered Arbitrable Data Auditing Scheme for Network Storage as a Service. IEEE Trans Serv Comput.

[58] Zhou J, Li L, Vajdi A, Zhou X, Wu Z (2021) Temperature-Constrained Reliability Optimization of Industrial Cyber-Physical Systems Using Machine Learning and Feedback Control. IEEE Trans Autom Sci Eng 1–12. 10.1109/TASE.2021.3062408

[59] Xu J, Li D, Gu W (2022). UAV-assisted Task Offloading for IoT in Smart Buildings and Environment via Deep Reinforcement Learning. Build Environ.

[60] Hamilton MJ (2022). Collective Computation, Information Flow, and the Emergence of Hunter-Gatherer Small-Worlds. J Soc Comput.

[61] Chen Y, Zhao F, Chen X, Wu Y (2022). Efficient Multi-Vehicle Task Offloading for Mobile Edge Computing in 6G Networks. IEEE Trans Veh Technol.

[62] Ma Y, Sun H, Chen Y, Zhang J, Xu Y, Wang X (2021). DeepPredict: A Zone Preference Prediction System for Online Lodging Platforms. J Soc Comput.

[63] Zhou J, Zhang M, Sun J, Wang T, Zhou X, Hu S (2022). DRHEFT: Deadline-Constrained Reliability-Aware HEFT Algorithm for Real-Time Heterogeneous MPSoC Systems. IEEE Trans Reliab.

[64] Chen Y, Xing H, Ma Z, et al (2022) Cost-Efficient Edge Caching for NOMA-enabled IoT Services. China Commun

